# Unusual Presentation and Outcome in Acute Cocaine Intoxication With Multiorgan Failure: A Case Report

**DOI:** 10.7759/cureus.39729

**Published:** 2023-05-30

**Authors:** Andrew G Kim, Kevin Watat, Harith Ghnaima, Majid Yavari, Supratik Rayamajhi

**Affiliations:** 1 Internal Medicine, Michigan State University, East Lansing, USA

**Keywords:** drug induced hepatitis, ischemic cardiomyopathy, acute decompensated systolic heart failure, vasospasm, acute ischemic cerebral infarction, ischemic cerebrovascular disease, ischemic stroke, diffuse cerebral edema, multiorgan system failure, acute cocaine intoxication

## Abstract

Cocaine overdose remains a significant public health concern worldwide, with potentially life-threatening consequences. The range of presentation can vary from mild autonomic hyperactivity to severe vasoconstriction, causing multiorgan ischemia and even death. In cases of high-dose intoxication, the presentation can be atypical. In this case report, we present a compelling case of a patient who initially presented with cardiac arrest and atypical signs. The patient made a remarkable recovery and returned almost to her baseline. This case provides valuable prognostic insight into the outcomes of severe multiorgan failure resulting from cocaine toxicity.

## Introduction

Cocaine overdose is a prevalent cause of acute intoxication-related emergency department visits worldwide [1]. This condition results in vasoconstriction, which leads to ischemic injury in multiple organs. However, the injury can be reversible following vasodilation [2, 3]. Here, we present a case of severe multiorgan failure resulting from acute cocaine intoxication. The patient needed intensive care unit admission for intubation, mechanical ventilation, and vasopressor support. After discharge and outpatient follow-up, the patient's organ function and residual disabilities almost fully recovered to their baseline. This case highlights that ischemic injuries caused by cocaine intoxication can spontaneously resolve, despite presenting as critical initially.

## Case presentation

A 29-year-old female patient with a history of anxiety disorder and polysubstance use disorder, including alcohol consumption, marijuana smoking, and occasional cocaine snorting over the past couple of years, was brought to the emergency department after experiencing a cardiac arrest. She had snorted an excessive amount of cocaine and had reported headaches and chest pain before losing consciousness. Upon the arrival of emergency medical services, she was in a pulseless electrical activity, but spontaneous circulation was returned after four rounds of cardiopulmonary resuscitation.

**Figure 1:**
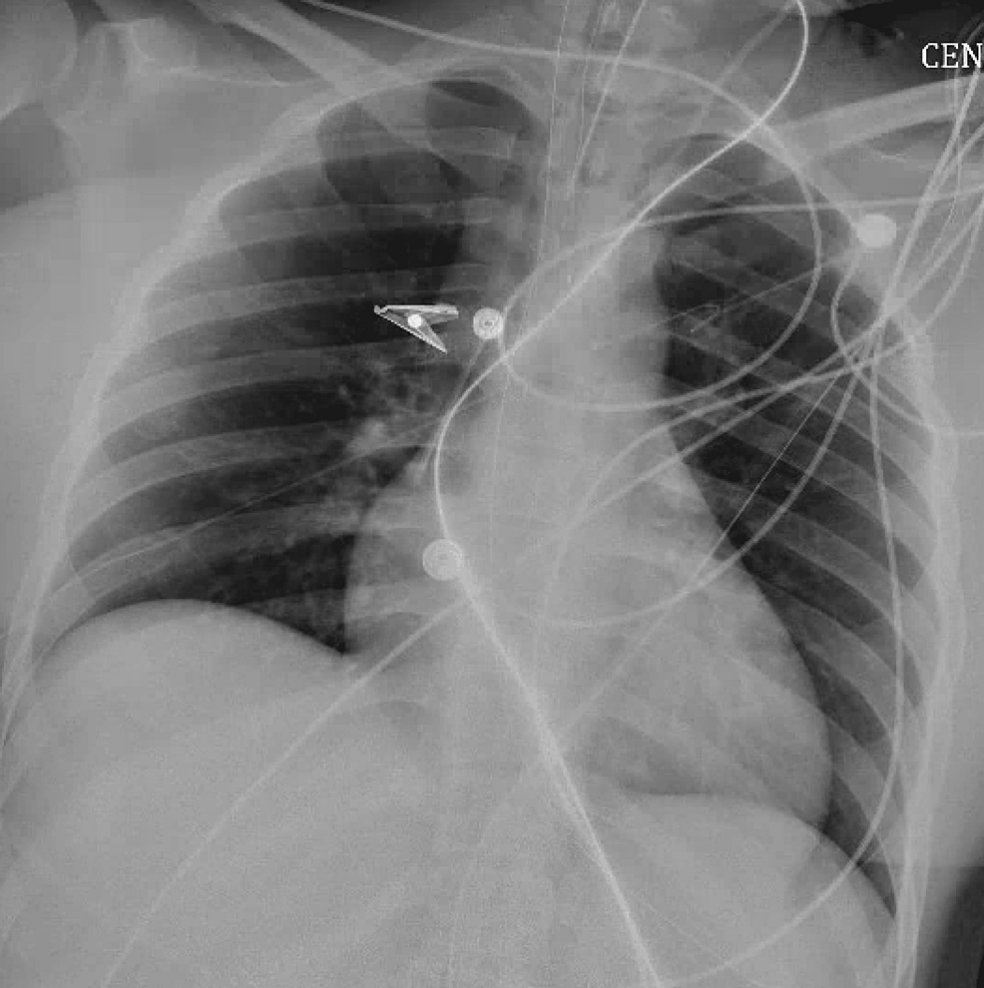
A chest X-ray taken shortly after the patient's arrival at the hospital, following endotracheal intubation and the insertion of a central line The chest X-ray showed no signs of acute abnormalities.

In the emergency department, she remained unresponsive, with vital signs of blood pressure 78/43 mmHg, heart rate of 96 beats per minute, and core body temperature of 90.7°F (32.6°C) on a warm summer day in August in Michigan. Her pupils were fixed at 3 mm and showed no reaction to light. The patient was intubated, mechanically ventilated, given intravenous fluid boluses, and transferred to the intensive care unit. The laboratory results showed an arterial pH of 7.19, lactate of 2.7 mmol/L, creatinine of 2.72 mg/dL, troponin I of 5190 ng/mL, creatine phosphokinase (CPK) of 5855 mcg/L, lactate dehydrogenase (LDH) of 4,219 IU/L, aspartate aminotransferase (AST) greater than 10,000 U/L, alanine transaminase (ALT) of 4600 U/L, and a positive urine drug screen for cocaine and benzoylecgonine consistent with cocaine intoxication. Cortisol levels were not evaluated, and microbiological cultures yielded negative results (Table 1, Figure 1).

**Table 1:** The initial laboratory values upon admission Significant results include troponin I high sensitivity of 5190 pg/mL, AST of >10,000 U/L, ALT of 4600 U/L, and creatinine of 2.72 mg/dL. WBC - white blood cell count, RBC - red blood cell count, MCV - mean corpuscular volume, MCH - mean corpuscular hemoglobin, MCHC - mean corpuscular hemoglobin concentration, RDW - red cell distribution width, MPV - mean platelet volume, BUN - blood urea nitrogen, PCO2 - partial pressure of carbon dioxide, PO2 - partial pressure of oxygen, O2 - oxygen, FiO2 - fraction of inspired oxygen, CPK - creatine phosphokinase, LDH - lactate dehydrogenase, AST - aspartate aminotransferase, ALT - alanine aminotransferase

Analysis	Result	Reference range
Complete blood count		
WBC	22.6	4.0 - 12.0 10^3/µL
RBC	3.89	3.50 - 5.55 10^6/µL
Hemoglobin	11.4	12.0 - 15.0 g/dL
Hematocrit	34.2	36.0 - 45.0 %
MCV	88	80 - 100 fL
MCH	29.3	27.0 - 33.0 pg
MCHC	33.3	31.0 - 37.0 g/dL
Platelet	41	150 - 400 10^3/µL
RDW	15.2	11.6 - 14.6 %
MPV	7.8	7.3 - 11.5 fL
Differential		
Neutrophils	93.6	49.0 - 81.0 %
Lymphocytes	4.8	14.0 - 41.0 %
Monocytes	1.5	0.0 - 11.0 %
Eosinophils	0	0.0 - 6.0 %
Basophils	0.1	0.0 - 3.0 %
Absolute neutrophils	21.1	1.96 - 9.72 10^3/µL
Absolute lymphocytes	1.1	0.56 - 4.92 10^3/µL
Absolute monocytes	0.3	0.00 - 1.32 10^3/µL
Absolute eosinophils	0	0.00 - 0.72 10^3/µL
Absolute basophils	0	0.00 - 0.36 10^3/µL
Chemical profile		
Sodium	141	135 - 145 meq/L
Potassium	4.8	3.5 - 4.9 meq/L
Chloride	112	96 - 110 meq/L
Bicarbonate	16	20.0 - 32.0 mmol/L
Anion gap	13	16-Feb
Glucose	85	65 - 99 mg/dL
BUN	35	6 - 23 mg/dL
Creatinine	2.72	0.60 - 1.40 mg/dL
Magnesium	2.2	1.6 - 2.5 mg/dL
Calcium	7.44	8.00 - 10.50 mg/dL
Ionized calcium	1.11	1.10 - 1.30 mmol/L
Phosphorus	6.7	2.5 - 4.5 mg/dL
Albumin	3	3.6 - 5.0 g/dL
Total protein	5.1	6.0 - 8.0 g/dL
Arterial blood gas		
pH	7.19	7.35 - 7.45
PCO2	35	34 - 46 mmHg
PO2	96	75 - 100 mmHg
Bicarbonate	12.6	20 - 30 mmol/L
Base excess	-14.6	
O2 saturation	96.5	95 - 98 %
FiO2	30%	
Cardiac profile		
CPK	5,855	0 - 155 U/L
LDH	4,219	100 - 225 U/L
Troponin I high sensitivity	5,190	0 - 18 pg/mL
B-natriuretic peptide	111	0 - 100 pg/mL
Liver profile		
AST	>10,000	10 - 40 U/L
ALT	4,600	3 - 45 U/L
Total bilirubin	0.5	0.2 - 1.2 mg/dL
Direct bilirubin	0.3	0.0 - 0.3 mg/dL
Alkaline phosphatase	82	37 - 98 U/L
Ammonia	123	13 - 37 µmol/L
Lactate	2.7	0.2 - 1.8 mmol/L
Urinalysis		
Type	Unspecified	
Color	Yellow	Yellow
Clarity	Slightly cloudy	Clear
Glucose	Negative	Negative
Ketones	Negative	Negative
Blood	Large	Negative
Nitrites	Positive	Negative
Leukocyte esterase	Negative	Negative
pH	6	4.5 - 8.5
Bilirubin	Negative	Negative
Urobilinogen	Negative	Negative
Urine microscopy		
WBC	10-Jun	0 - 5
RBC	20-Nov	0 - 3
Bacteria	Few	None
Squamous cells	Occasional	
Urine toxicology		
Barbiturates	Negative	200 ng/mL
Cannabinoids	Negative	20 ng/mL
Cocaine	Positive	300 ng/mL
Opiates	Negative	300 ng/mL
Phencyclidine	Negative	25 ng/mL
Methadone	Negative	300 ng/mL
Benzodiazepines	Negative	200 ng/mL
Benzoylecgonine	Positive	30 ng/mL
Amphetamines/methamphetamines	Negative	1000 ng/mL
Tricyclinc antidepressants	Negative	300 ng/mL
Oxycodone	Negative	50 ng/mL
Ecstasy	Negative	500 ng/mL
Specimen validation pH	6.5	
Specimen validation specific gravity	1.025	

Despite the patient experiencing an initial out-of-hospital cardiac arrest, targeted temperature management to induce therapeutic hypothermia was not initiated due to the patient already having been hypothermic for the initial 24 hours. Additionally, therapeutic alpha-blockers to address cocaine-induced vasoconstriction were not administered due to severe hypotension. Initially, the patient was completely unresponsive without any sedation, but later sedation was started when the patient began exhibiting autonomic hyperactivity.

**Figure 2:**
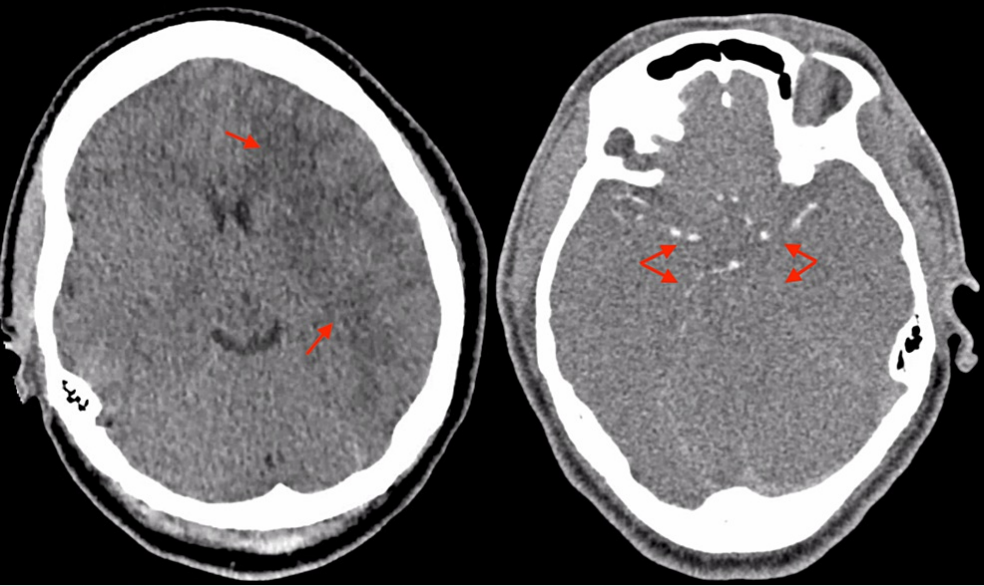
The CT and CT angiogram of the head upon admission Left: The CT scan of the head revealed infarction in the middle cerebral artery territory, with the left hemisphere (Arrows) affected more than the right. Right: The CT angiogram revealed opacification of the bilateral proximal middle cerebral arteries (upward arrows) and posterior cerebral arteries (downward arrows).

The initial CT and CT angiogram of the brain showed diffuse cerebral edema and multiple infarctions in the bilateral middle and posterior cerebral arteries, most prominent in the left middle cerebral artery territory on the CT scan (Figure 2). The initial echocardiogram showed a left ventricular ejection fraction of less than 20%, with diffuse severe hypokinesis and akinesis. The patient's renal function continued to decline for the first three days, and continuous renal replacement therapy was initiated. Later, when the patient's hemodynamic status improved, a tunneled dialysis catheter was inserted to begin hemodialysis.

**Figure 3:**
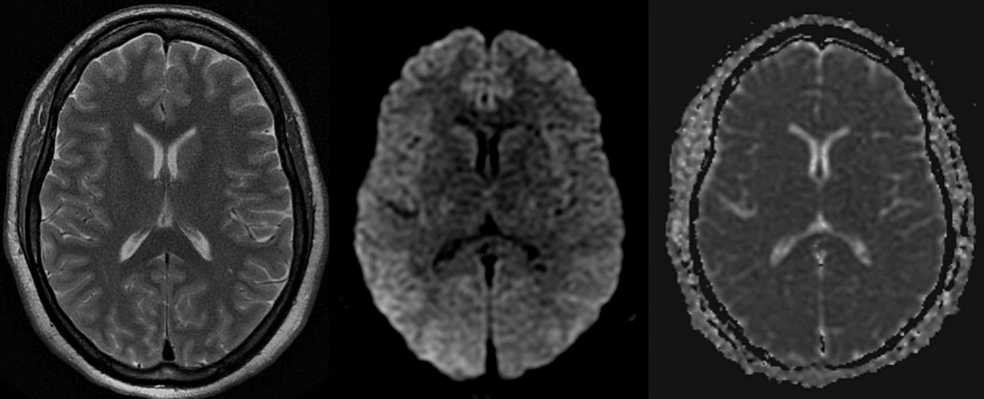
Follow-up MRI of the brain on day four The initial findings of diffuse cerebral edema and infarctions, as depicted in Figure 2, have resolved. Left: T2-weighted image; Middle: diffusion-weighted image; Right: apparent diffusion coefficient image

However, on the follow-up echocardiogram on day three, the ejection fraction significantly improved to 70-75%, and the wall motion abnormalities resolved spontaneously. On day four, an MRI showed that the diffuse cerebral edema and multiple infarctions in the cerebral arteries visible on the initial CT scan had spontaneously resolved (Figure 3). After being extubated, the patient exhibited significant weakness in her right arm, likely as a result of a temporary infarction in the left middle cerebral artery. However, this focal weakness fully resolved over time with physical therapy. Despite initial severe shock liver, the patient's liver function tests had also rapidly improved without signs of decompensation.

The patient's acute kidney injury persisted in the context of shock kidneys and rhabdomyolysis. On day 17, the patient was discharged with a plan to receive scheduled outpatient hemodialysis for the ongoing treatment of her acute kidney injury. However, during the follow-up, the patient's acute kidney injury had completely resolved as well, and the dialysis catheter was removed.

## Discussion

Cocaine inhibits the neuronal uptake of norepinephrine, which in turn stimulates adrenergic receptors, leading to vasospasm and sympathomimetic overstimulation [2, 3]. It also blocks sodium channels and possesses local anesthetic properties [4].

Cocaine can lead to ischemic organ failure either through cardiogenic shock [5] or by inducing direct vasospasm of the vasculature supplying the target organ [6, 7]. Stimulation of alpha-adrenergic receptors of coronary artery smooth muscle cells induces coronary artery vasospasm and vasospastic angina [8], which the patient likely experienced before cardiac arrest. Nevertheless, cocaine can occasionally result in acute ischemia in different organs by causing thromboembolism as well [9].

Typical signs of cocaine overdose include hypertension, tachycardia, and hyperthermia. However, clinicians need to be cautious because, in cases of high-dose intoxication, patients may present with the opposite signs of hypotension and bradycardia due to severe ischemic multiorgan failure [10].

Hypothermia is a rare occurrence in patients with cocaine overdose, particularly during the summer. Although animal studies have demonstrated that cocaine can induce hypothermia [11], it has rarely been reported in human subjects. Fuller et al. (2008) also presented a patient who experienced initial hypothermia and cardiac arrest due to massive cocaine intoxication, but therapeutic hypothermia was induced to further lower the patient's body temperature. Similar to our case, this patient had nearly complete neurological recovery [12]. In order to determine the extent of this phenomenon, it is considered essential to have more case reports or case series that involve human subjects. Therapeutic hypothermia is often utilized in patients who experience cardiac arrest, and the initial hypothermia observed in our case likely contributed to the positive outcome [13]. 

## Conclusions

Acute cocaine intoxication commonly presents with signs of hypertension, tachycardia, and hyperthermia. However, high-dose intoxication may result in atypical symptoms like hypotension, bradycardia, and hypothermia, as demonstrated in this case. Hypothermia in humans with cocaine overdose is rarely reported and requires further investigation. Cocaine can cause severe ischemic multiorgan failure and even death. Nevertheless, vasospasm triggered by cocaine use is reversible, which might explain why the prognosis for the patient with severe ischemic multiorgan failure due to cocaine overdose was surprisingly favorable when compared to other causes.
